# A comparative analysis of CNN-based deep learning architectures for early diagnosis of bone cancer using CT images

**DOI:** 10.1038/s41598-024-52719-8

**Published:** 2024-01-25

**Authors:** Kanimozhi Sampath, Sivakumar Rajagopal, Ananthakrishna Chintanpalli

**Affiliations:** 1 Department of Sensor and Biomedical Technology, School of Electronics Engineering, Vellore Institute of Technology, Vellore, 632014 India; 2 Department of Communication Engineering, School of Electronics Engineering, Vellore Institute of Technology, Vellore, 632014 India

**Keywords:** Bone cancer, Diagnosis, Medical imaging

## Abstract

Bone cancer is a rare in which cells in the bone grow out of control, resulting in destroying the normal bone tissue. A benign type of bone cancer is harmless and does not spread to other body parts, whereas a malignant type can spread to other body parts and might be harmful. According to Cancer Research UK (2021), the survival rate for patients with bone cancer is 40% and early detection can increase the chances of survival by providing treatment at the initial stages. Prior detection of these lumps or masses can reduce the risk of death and treat bone cancer early. The goal of this current study is to utilize image processing techniques and deep learning-based Convolution neural network (CNN) to classify normal and cancerous bone images. Medical image processing techniques, like pre-processing (e.g., median filter), K-means clustering segmentation, and, canny edge detection were used to detect the cancer region in Computer Tomography (CT) images for parosteal osteosarcoma, enchondroma and osteochondroma types of bone cancer. After segmentation, the normal and cancerous affected images were classified using various existing CNN-based models. The results revealed that AlexNet model showed a better performance with a training accuracy of 98%, validation accuracy of 98%, and testing accuracy of 100%.

## Introduction

Bones are made of two regions, outer and inner regions. The outer region is compact and enclosed by cancellous tissues while the inner region consists of blood-producing material^1^. Bone cancer can originate from any part of the bones and can occur due to hereditary factors or previous radiation exposure. The benign cancer occurs commonly and is asymptomatic until the disease spreads or injuries the other body parts. The malignant cancer can lead to the patient’s death unless treated at the early stage^2^. Since most of the cancers are asymptomatic, early diagnosis and treatment is critical to stop spreading to the other regions of the body. Bone cancer is divided into primary and secondary types. If the unrestricted cell growth is not treated during the primary type, cancer can develop unwanted new cells which may later lead to death. In the primary type, cancer starts from cells of bone whereas in the secondary type, cancer starts from other body regions and then affect the cells of the bone^3^. Primary detection of bone cancer has a chance of reducing the death rate. In the beginning stage, the symptoms of bone cancer may include bowel movement change, formation of new lumps, weight loss, bone loss, pain and, weakness in bones^4^. Proper treatment of cancer requires information like the history of patients, physical examination, and imaging techniques (e.g., X-ray^2^, Computed Tomography (CT)^5^, Magnetic Resonance Imaging (MRI)^6^, and Positron Emission Tomography (PET)^7^). Radiologists prefer medical imaging procedure for the detection of cancer due to the management of time, low cost and early detection. The preprocessing, segmentation, feature extraction, and classification stages are incorporated in medical devices for early diagnosis^8^. Moreover, the pre-processing stage includes, either bilateral, median or Gaussian filter to remove the noise from the images^9,10^. After the noise removal, cancer regions can be segmented either using the threshold based^11^, region based^11,12^ or edge based segmentation^13^ methods. The segmentation techniques like Prewitt, Canny, Sobel, K-means and region growing were used to analyze the osteosarcoma type of bone cancer in X-ray images^2,10,13^. The K-means and edge detection segmentation algorithms have also been used for bone cancer^14^. After segmenting the cancer regions, seven Gray Level Co-occurrence Matrix (GLCM) features were extracted from the image. These features were then trained and tested using the K-nearest neighbors (KNN) classifier with a resulting accuracy of 98.18%^14^. The fusion of K-means with the fuzzy C-means segmentation of the MRI images was used to calculate the mean intensity to identify the cancer and non-cancer images. The accuracy rate was 98% with a sensitivity of 65.21% and a specificity of 98.47%^15^. The X-ray images of 105, with 65 cancers and 40 normal, were used to extract the histogram of the gradient with GLCM features. Using the support vector machine (SVM) classifier, an accuracy of 92.5% was achieved^16^. The 36 X-ray images were used to extract the cancer border clarity and GLCM features and these features were then used to classify the benign and cancerous image using random forest and SVM classifiers with the resulting testing accuracy of 85% and 81%, respectively. Among these two classifiers, random forest performed well compared to SVM which may be due to the use of small dataset and decision tree in a random forest classifier whereas SVM uses only the linear kernel, hence random forest works faster and performs good result^17^. Recently, the development of Artificial Intelligence (AI) has becoming more advanced in medical image analysis^18–20^. Deep neural networks (DNNs) are used as computational models to acquire training to learn the features of the images from a large set of datasets, resulting in reduction of false positive and false negative rates and thereby increasing the accuracy rate during the testing stage^20,21^. The previous works on DNN primarily focused on X-ray^2,9^ and MRI images^2,22,23^ for bone cancer diagnosis while usage of CT images is rare due to the limited numbers of publicly available database^5,24,25^. The 2899 X-ray images were used to evaluate the 3 way classification (benign, intermediate and malignant) using Convolutional neural network (CNN) classifier and achieved the testing accuracy of 73.4%^9^. To classify the normal and bone cancer images, the 1060 MRI images were divided into training (70%), validation (20%) and testing (10%). EfficientNet B0 was then used for the image classification and achieved the testing accuracy of 72%^6^.The 39 MRI images with histopathological confirmation were used to predict the malignancy in the bone cancer using DNN. The dataset were splitted into training (70%), validation (10%), testing (20%) and then ResNet50 model was used to classify the benign and malignant type of bone cancer with the resulting testing accuracy of 95%^23^. The 832 CT scans, with 732 for training, 40 for validation and 60 for testing, were used to segment and classify the cancer regions using 2D and 3D UNet model and 3D ResNet, respectively. This model achieved the testing sensitivity of 82.7% with 0.617 false positive rate^5^.

The Computer aided design (CAD) system were presented to distinguish the benign and malignant type of bone cancer in 79 CT images. Active contour model were used to segment the cancer regions and then GLCM features were extracted to train and test using the Random Forest classifier and obtained the overall testing accuracy of 91.47%^24^. The K-mean clustering segmentation algorithm was used to segment the cancer regions in 3 MRI and 3 CT images. The surface area of the cancer regions were evaluated using the algorithm and compared with the radiologist performance. The relative difference of algorithm and radiologists ranges from 0.63 to 1.75% for MRI images and 0.34 to 1.51% for CT images^25^. As CT is the primary scan after X-ray, hence is necessary to conduct a thorough investigation using the CT scans for detecting early bone cancer. Usually, CT scans preferred over other medical imaging modalities due to the excellent spatial resolution and lesser scanning time^12^. CT is also the best imaging method to visualize the complex bone structures in the early stage for detecting the bone metastasis^12,26^. The current study deals with commonly affected bone cancers for the early detection of parosteal osteosarcoma^27^, enchondroma^28^, and osteochondroma^29^ types of bone cancer. Perosteal osteosarcoma is the primary malignant type which arises on the surface of the bone^30^. The common location is metaphyseal to diaphyseal junction or the diaphysis part of the long bone like humerus, tibia, mandible, and femur^31^. Enchondroma commonly occurs in the cartilage inside the bone^32^ and osteochondroma occurs in the end of growth plate of long bone^33^. The goal of this study is to detect bone cancer at a preliminary stage by utilizing the larger datasets of CT images and applying the image processing and deep learning (DL) techniques to detect the cancer with higher accuracy rate. More specifically, using 1141 bone CT images, the current study utilized K-means clustering, canny edge detection segmentation, and CNN models to classify the normal and cancerous images.

## Methods

**Figure 1:**
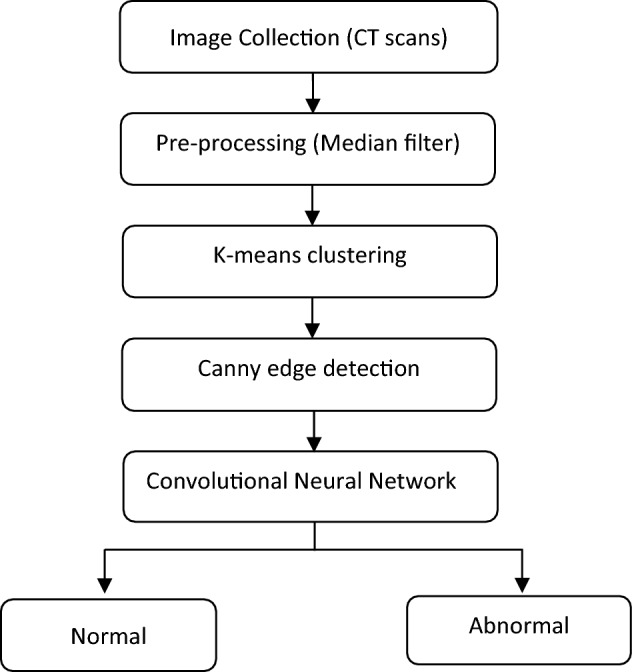
Flowchart illustrating the steps involved in the detection of bone cancer.

The proposed method involved detection and classification of bone cancer. The cancer region has more intensity than the other regions in the image^24,34^. Figure 1 shows the flowchart of the step involved in detecting the cancer region from the CT image for classifying the normal and cancer affected bones.

### Image collection

The bone cancer images are obtained from publicly available databases: radiopeadia (radiopeadia.org) and cancer_imaging_archive (cancerimagingarchive.net). The dataset used in this study consists of 1141 CT scan images (730 CT scans from radiopeadia and 411 CT scans from cancer_imaging_archive), with 530 bone cancer images and 511 normal images.

### Pre-processing

The image was converted into a grayscale prior to applying the filter^34^. There exists many filters (e.g., Average, Median, Gaussian, Weiner filters) for noise reduction during the pre-processing stage^25^. Among these, the median filter had a better performance for early-stage detection of the bone cancer images^24^. Moreover, this is a non-linear method that is effective in removing the salt and pepper noise while preserving the edges^25,34^.

### Image segmentation using K-means clustering

K-means clustering is the unsupervised learning^35^ to classify the data into clusters (or groups). In the K-means clustering algorithm, the number of clusters (e.g., $k$) is required to be known. Initially, ‘$k$’ centroids are selected randomly in the dimensional space. The squared Euclidean distance metrics were computed between each data point and all the centroid locations. The minimum distance is then used to cluster the data to a specific centroid. The location of each centroid is updated by averaging all the data points that belong to a specific cluster. This procedure of computing the distance metric and updating the centroid location is repeated until there is no change in centroid location^35,36^. This algorithm was mainly used to segment the cancer region from the original CT image.

### Canny edge detection

The edge detection is used to find the object boundaries by detecting the discontinuities in the image. This is widely applied in the image processing applications for extracting relevant features from an image^37^. Different types of edge detection techniques are Sobel, Prewitt, Roberts, and Canny^10,15,35^. Among these, the canny edge detection method provides better results for early-stage detection of bone cancer but this technique requires thresholding-in which low and high threshold values are chosen based on the histogram of the images^35^. Moreover, this approach performs well compared to other edge detection methods due to specific advantages: localization of edges, reduction of noise and gradient information^37^.

Canny edge detection consists of a Gaussian filter, gradient magnitude, non-maxima suppression and two threshold values. This approach has a single response and better localization to accurately identify weak and strong backgrounds without missing any detail information^36^.The gradient magnitude can be calculated by using^13,36^:

$${G}_{x}=\left[\begin{array}{ccc}1& 0& 1\\ -2& 0& 2\\ -1& 0& 1\end{array}\right]\times A\quad {G}_{y}=\left[\begin{array}{ccc}1& 2& 1\\ 0& 0& 0\\ 1& -2& -1\end{array}\right]\times A,$$

$$\left|G\right|=\sqrt{{G}_{x}^{2}+{G}_{y}^{2}},$$

$$Angle \left(\theta \right)={\text{tan}}^{-1 }\frac{{G}_{y}}{{G}_{x}},$$

where ${G}_{x}$ represents horizontal edges, ${G}_{y}$ represents vertical edges, and $A$ represents the filtered bone cancer image that convolves with the 3 × 3 convolutional kernel to detect the horizontal and vertical edges. The non-maxima suppression is used to narrow the edges of the image. If the gradient of the pixel is lesser than the lower threshold value, then the pixel is neglected and if the gradient of the pixel is greater than the higher threshold value, the pixel is accepted^36^. If the gradient of the pixel lies between lower and upper threshold value and the pixel is connected to edge, then only the pixel is accepted^10,36^.

### Convolutional neural network

**Figure 2:**
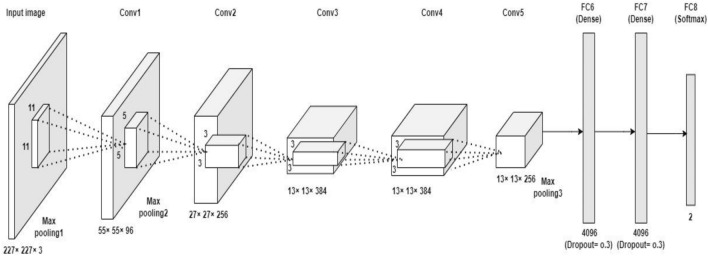
The AlexNet architecture for detecting normal and cancerous CT bone images^38,40^.

Convolutional Neural Network (CNN) is commonly used for classifying the medical images with good accuracy and better performance^36,38,39^ The CNN is a supervised learning scheme that processes the input images and produces the output to determine whether the disease exists or not. The current study had utilized AlexNet model as shown in Fig. 2. This network architecture consists of eight layers; the first five were convolutional layers with the combination of maxpooling and next 3 were fully connected layers^36,38^. After each convolutional layers, a rectifier linear unit (ReLU) activation function is used. The convolutional layers utilize specific number of filters (along with ReLU) to extract the relevant features from the input image. The maxpooling layer (an optional layer), is then used to remove the computational complexity while preserving the features. Followed by convolutional and pooling layers, there are 3 fully connected layers that flatten the features of the image. A dropout layer exists between fully connected layer to prevent the over fitting problems. The last layer is the fully connected layer that uses softmax activation function to analyze the probabilities of each class^36,38–40^. The layer specifications like filter size, kernel size, stride, input shape and output shape of the AlexNet architecture is shown in Table 1.

**Table 1:** Layer specifications of the AlexNet architecture^38,40^.

Layer	Filter size	No. of filters	Stride	Input dimension	Output dimension	Activation function
Convolution 1	11 × 11	96	4	227 × 227 × 3	55 × 55 × 96	ReLU
Maxpooling	3 × 3	–	2	55 × 55 × 96	27 × 27 × 96	–
Convolution 2	5 × 5	256	1	27 × 27 × 96	27 × 27 × 256	ReLU
Maxpooling	3 × 3	–	2	27 × 27 × 256	13 × 13 × 256	–
Convolution 3	3 × 3	384	1	13 × 13 × 256	13 × 13 × 384	ReLU
Convolution 4	3 × 3	384	1	13 × 13 × 384	13 × 13 × 384	ReLU
Convolution 5	3 × 3	256	1	13 × 13 × 384	13 × 13 × 256	ReLU
Maxpooling	3 × 3	–	2	13 × 13 × 256	6 × 6 × 256	–
Flatten	–	–	–	6 × 6 × 256	9216	–
Dense	–	–	–	9216	4096	ReLU
Dense	–	–	–	4096	4096	ReLU
Dense (output)	–	–	–	4096	2	Softmax

In the current study, various CNN models like AlexNet^41^, ResNet50^42^, ResNet101^43^, VGG16^43^, VGG19^43^, InceptionV3^42^, Xception^44^, DenseNet121^42,43^, EfficientNet B0^6^ and EfficientNet B2^45^ were applied to classify the CT image either into normal or cancer. Each CNN model was trained to perform two-way classification (normal and malignant). The input image size, number of epochs, loss function, and learning optimizer were the same for all the CNN models to facilitate the comparison in terms of accuracy and computational processing time. The size of the input image was 227 × 227 and the batch size was set to 32. Adam optimizer was used with the learning rate of 0.001, due to its better convergence, less memory requirements and computationally efficient compared to Stochastic and RMSprop optimizers^46^. Since the model focuses on two way classification, binary cross entropy loss function^47^ was used for all CNN models during the training, validation and testing stages. These models were implemented in Python using Jupyter Notebook version 6.4.12. The accuracy of the classification model was calculated using the equation:-

$$Accuracy=\frac{(TP+TN)}{(TP+TN+FP+FN)},$$

where TP represents the true positive rate (i.e., diseased images are correctly predicted as diseased images), FP represents the false positive rate (i.e., normal images are wrongly predicted as diseased images), FN represents the false negative rate (i.e., diseased images are wrongly predicted as normal images) and TN represents the true negative rate (i.e., normal images are correctly predicted as normal images)^48,49^.

## Results and discussion

**Figure 3:**
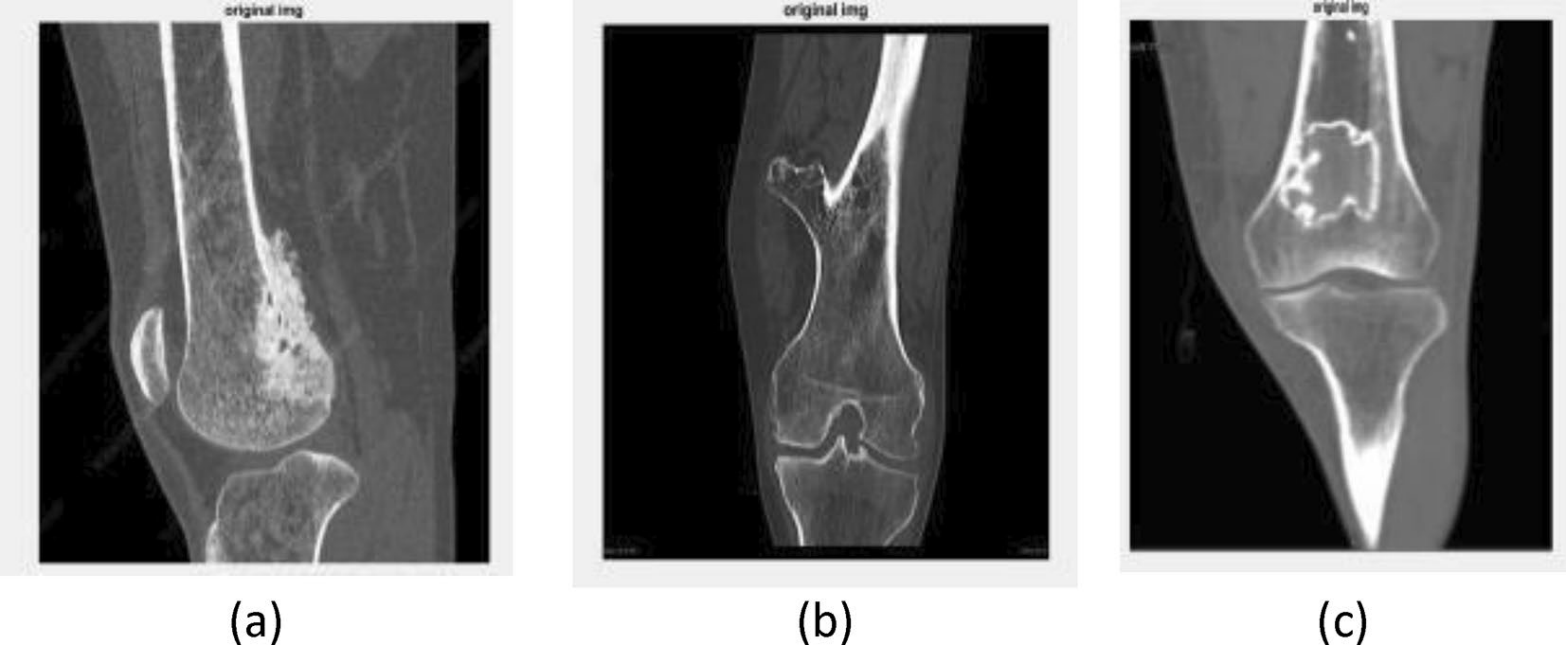
Original CT images: (**a**) lateral CT of parosteal osteosarcoma, (**b**) coronal CT of Osteochondroma, and (**c**) lateral CT of Enchondroma.

The CT images of Parosteal osteosarcoma, Osteochondroma and Enchondroma types of bone cancer images were used for analysis in the current study and are shown in Fig. 3.

**Figure 4:**
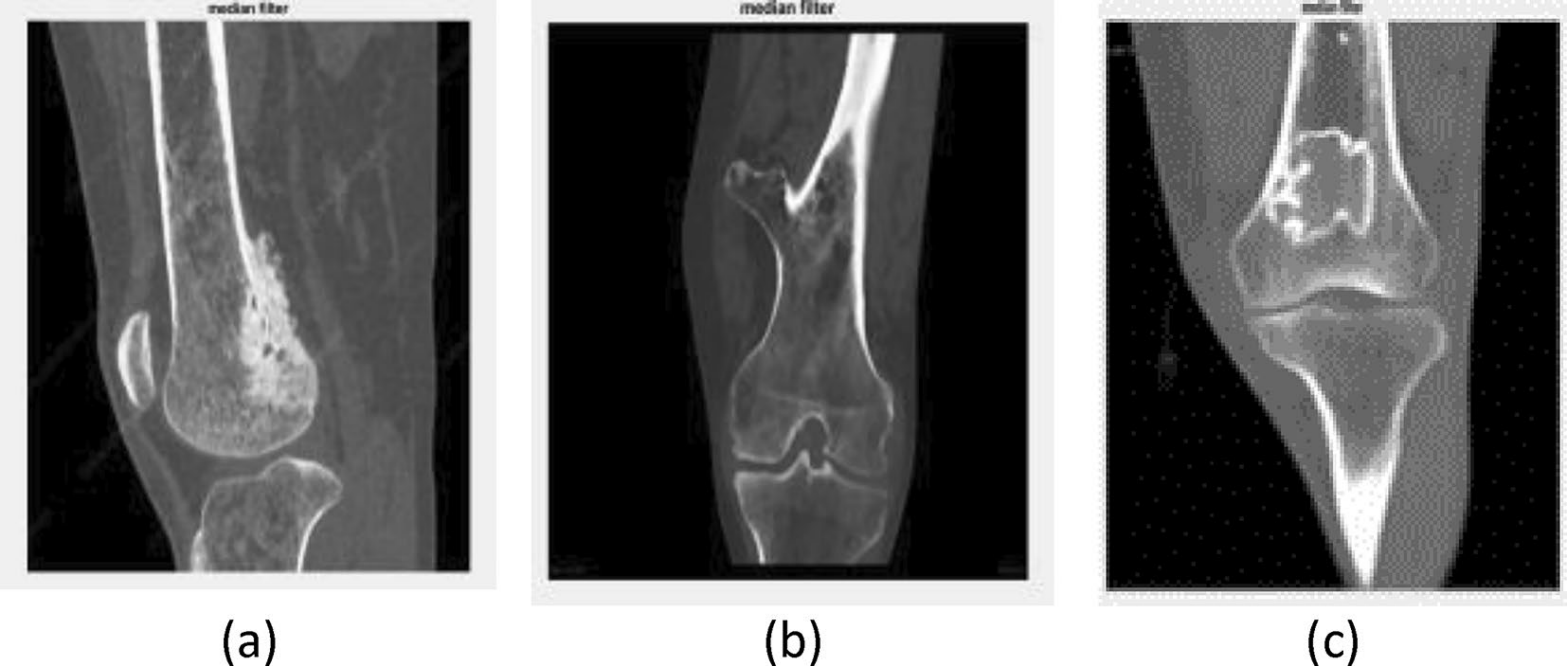
Effect of the median filter: (**a**) lateral CT of parosteal osteosarcoma, (**b**) coronal CT of Osteochondroma, and (**c**) lateral CT of enchondroma.

**Figure 5:**
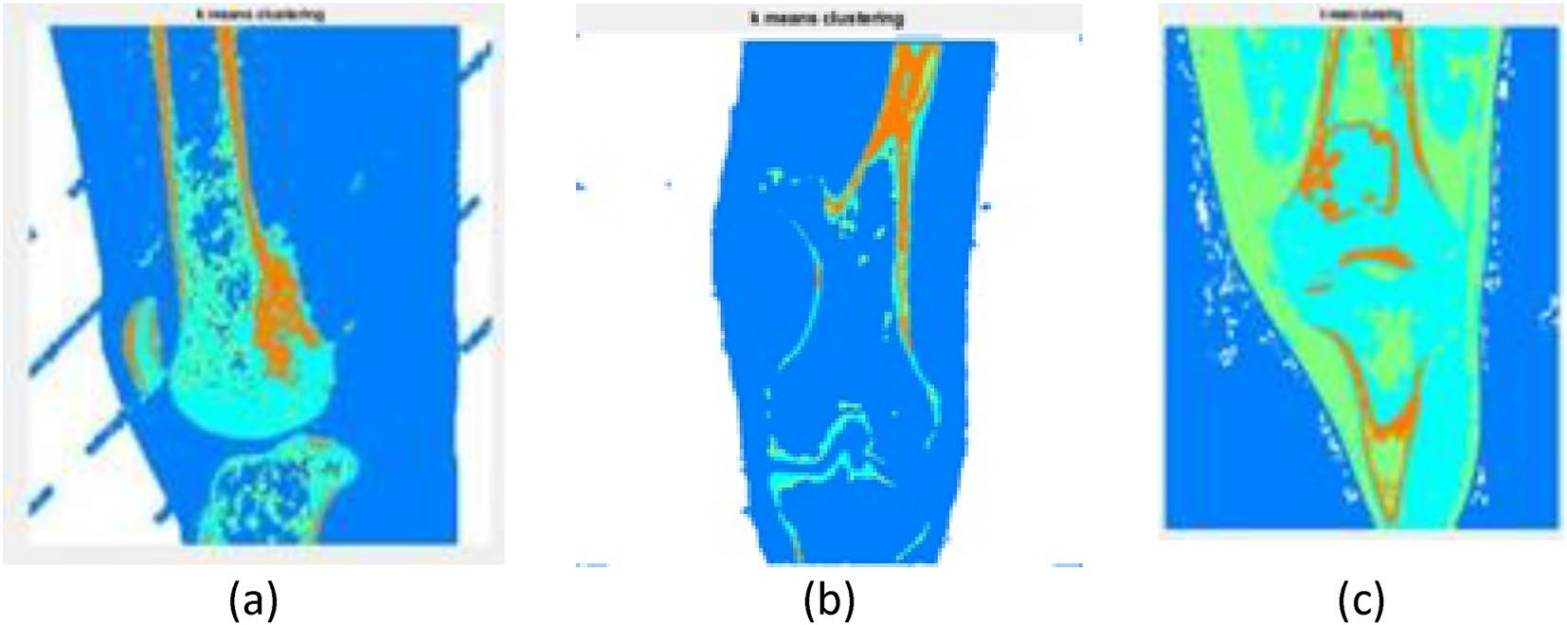
Effect of K-means clustering: (**a**) lateral CT of Parosteal osteosarcoma, (**b**) coronal CT of osteochondroma, and (**c**) lateral CT of enchondroma.

**Figure 6:**
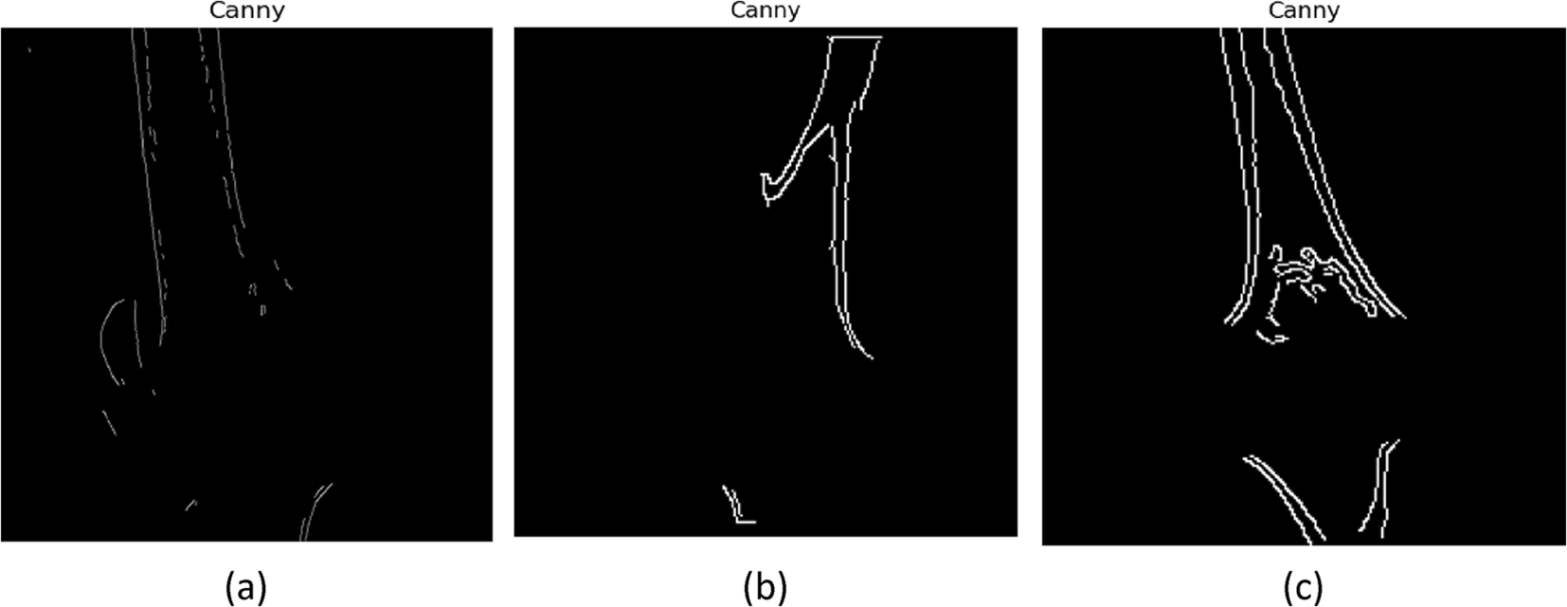
Canny edge detection: (**a**) lateral CT of parosteal osteosarcoma, (**b**) coronal CT of osteochondroma, and (**c**) lateral CT of enchondroma.

Figure 4 describes the filtered CT images after the median filter. The original CT images (as shown in Fig. 3) usually contain noise that reduces the visibility of the low—contrast pixels in the image. Thus, the noise present in Fig. 4 has been removed using the median filter to increase the contrast of the images. The K-means clustering segment the filtered CT image into different regions based upon pixel intensity which aids to identify the area that contain cancerous growth. More specifically, the red colour label in Fig. 5 represents the bone cancer-affected region. Figure 6 describes the segmented edges and boundaries of the cancer affected area after applying the canny edge detection algorithm.

**Figure 7:**
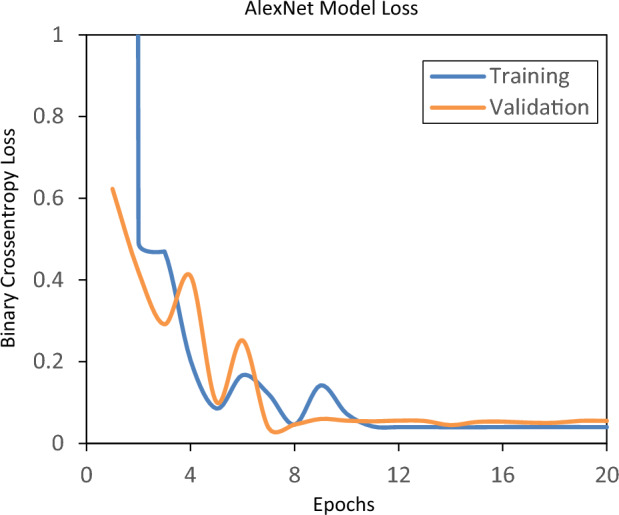
Total weighted loss of AlexNet model during training and validation stages.

**Figure 8:**
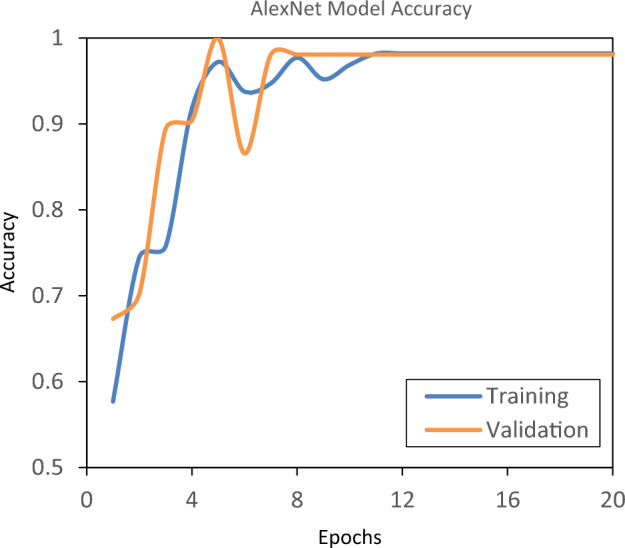
Accuracy of AlexNet model during training and validation stages.

The dataset was divided into 80% for training, 10% for validation, and 10% for testing. Figures 7 and 8 depict the graphical representation of binary cross entropy loss and accuracy of AlexNet model. As shown in Fig. 7, at the initial epoch value the total weighted loss was high and then the loss was decreased as the epoch value was increased. The accuracy, as shown in Fig. 8, was lower at the initial epoch value and then improved with increasing epoch value. From epoch 14 onwards (Fig. 7), the training and validation losses converge, indicating that the training can be stopped. For comparative analysis across various CNN-based models, the epoch number was selected when any one of the models reached 100% accuracy during the testing stage. In this case, AlexNet reached 100% accuracy at 20th epoch and hence number of epoch was set to 20 for all the CNN models.

Table 2 describes the results of two way classification performed by AlexNet, ResNet50, ResNet101, VGG16, VGG19, DenseNet121, EfficientNet B0, EfficientNet B2, Xception, and InceptionV3 models. Among these models, AlexNet performed well with the training accuracy of 98%, validation accuracy of 98% and testing accuracy of 100% with lesser computational processing time (29 min) compared to other CNN models.

**Table 2:** Comparison performance of each convolutional neural network (CNN) model.

Classification model	Training accuracy (%)	Validation accuracy (%)	Testing accuracy (%)	Computational processing time (min)	Number of epochs
AlexNet	98	98	100	29	20
ResNet50	84	83	81	50	20
ResNet101	88	92	89	71	20
VGG16	83	77	74	120	20
VGG19	86	87	80	150	20
DenseNet121	64	64	68	33	20
EfficientNet B0	86	94	89	17	20
EfficientNet B2	87	91	91	48	20
Xception	65	58	68	105	20
InceptionV3	59	59	69	51	20

## Conclusion

Bone cancer is one of the hazardous disease and hence early detection is utmost important for better diagnosis. This can be diagnosed based on three elements: symptoms, histopathological and imaging. The symptoms are mostly nonspecific during the initial stages whereas histopathology examination is an invasive method that detects the cancer mostly at the final stage but not during initial stage. In such cases, imaging has the ability to differentiate the normal and cancerous image during the early stage. The goal of this current study is to detect and classify bone cancer present in the CT images using various image processing techniques along with the various CNN models. The image processing techniques were used to detect the cancer region using pre-processing (median filter) to remove the noise in the image, K- means clustering to segment the cancer region, canny edge detection segmentation to extract the cancer edges. When compared with other CNN models, the AlexNet model showed the best performance, with training accuracy of 98%, validation accuracy of 98%, testing accuracy of 100% and lowest computational processing time. Thus, AlexNet could be a useful tool to predict the bone cancer at the early stage from CT images using DNN. As a future work, the low, medium, and high level features from the CT images can also be extracted prior to classification using DNNs (e.g., ResNet, VGGNet and DenseNet) to achieve automated AI based model to detect the stages of bone cancer and classification of normal and subtypes of bone cancer.

## Data Availability

The dataset generated and/or analyzed during the current study are available in the [radiopeadia and cancerimagingarchive] repositories, [www.radiopeadia.org and www.cancerimagingarchive.net].
